# IGF-1-releasing PLGA nanoparticles modified 3D printed PCL scaffolds for cartilage tissue engineering

**DOI:** 10.1080/10717544.2020.1797239

**Published:** 2020-07-25

**Authors:** Peiran Wei, Yan Xu, Yue Gu, Qingqiang Yao, Jiayin Li, Liming Wang

**Affiliations:** a Department of Orthopaedics, Nanjing First Hospital, Nanjing Medical University, Nanjing, China; b Key Lab of Additive Manufacturing Technology, Institute of Digital Medicine, Nanjing Medical University, Nanjing, China; c Cartilage Regeneration Center, Nanjing First Hospital, Nanjing Medical University, Nanjing, China

**Keywords:** IGF-1, nanoparticles, PCL, 3D-printed, cartilage tissue engineering

## Abstract

The aim of this study is to fabricate and test a 3D-printed PCL scaffold incorporating IGF-1-releasing PLGA nanoparticles for cartilage tissue engineering. IGF-1 loaded PLGA nanoparticles were produced by the double-emulsion method, and were incorporated onto 3D printed PCL scaffolds via PDA. Particle size, loading effciency (LE) and encapsulation effciency (EE) of the nanoparticles were examined. SEM, pore size, porosity, compression testing, contact angle, IGF-1 release kinetics of the composite scaffolds were also determined. For cell culture studies, CCK-8, Live/dead, MTT, GAG content and expression level of chondrocytes specific proteins and genes and HIF-1α were also tested. There was no difference of the nanoparticle size. And the LE and EE of IGF-1 in PLGA nanoparticles was about 5.53 ± 0.12% and 61.26 ± 2.71%, respectively. There was a slower, sustained release for all drug-loaded nanoparticles PLGA/PDA/PCL scaffolds. There was no difference of pore size, porosity, compressive strength of each scaffold. The contact angles PCL scaffolds were significant decreased when coated with PDA and PLGA nanoparticales. (*p* < .05) Live/dead staining showed more cells attached to the IGF-1 PLGA/PDA/PCL scaffolds. The CCK-8 and MTT assay showed higher cell proliferation and better biocompatibility of the IGF-1 PLGA/PDA/PCL scaffolds. (*p* < .05) GAG content, chondrogenic protein and gene expression level of SOX-9, COL-II, ACAN, and HIF pathway related gene (HIF-1α) were significantly higher in IGF-1 PLGA/PDA/PCL scaffolds group compared to other groups. (*p* < .05) IGF-1 PLGA/PDA/PCL scaffolds may be a better method for sustained IGF-1 administration and a promising scaffold for cartilage tissue engineering.

## Introduction

As cartilage is a tissue with poor capacity of regeneration, cartilage tissue engineering offers the ability to repair cartilage by combining cells, scaffolds, and signals (Armiento et al., 2018; Li et al., 2019). Insulin‐like growth factor‐1 (IGF‐1) has been extensively researched for its ability to encourage cell proliferation, inhibition of cell apoptosis, and anabolic effects on musculoskeletal tissues (Huat et al., 2014; Ramos et al., 2019). It has been demonstrated that IGF-1 is an anabolic growth factor that is very important in cartilage development and homeostasis (Tuncel et al., 2005). And Many studies have demonstrated the efficacy of IGF-1 for articular cartilage repair, but it is dose-dependent (Tuncel et al., 2005; Mullen et al., 2010). Thus, sustained release of IGF-1 is required.

Polylactic acid-glycolic acid (PLGA), has now been widely used as a drug carrier in tissue engineering applications, which can be used to fabricate drug-releasing nanoparticles (Martins et al., 2018). With its adjustable degradation characteristics and biocompatibility, PLGA nanoparticles can be fabricated to deliver almost all types of drugs (Wang et al., 2011).

It is known that biomimetic porous scaffolds with interconnected porosity play an essential role in tissue engineering (Zhou et al., 2011; Wu et al., 2014). Currently, 3D printing allows fabrication of 3D scaffolds of virtually any shape from a computer aided design (CAD) for tissue engineering (Cox et al., 2015). Poly-ɛ-caprolactone (PCL) is a promising material used in 3D printing in the field of tissue engineering (Malikmammadov et al., 2018), due to its superior mechanics, biodegradability, bioactivity, and material processability (Makris et al., 2011). However, PCL is known for its hydrophobic nature, which can result in decreased cell‐material interaction (Li et al., 2019; Ramos et al., 2019). Polydopamine (PDA) coating is widely used and easily prepared. Surface modification with PDA helps improve hydrophilicity and cellular performance of PCL scaffolds, such as cell adhesion, proliferation, and chondrogenetic differentiation, while maintaining or even improving the physical properties and structure (Li et al., 2019), and can form a tightly adhered coating on the surface of many organic or inorganic materials (Zhou et al., 2018; Ho & Ding, 2014).

Given this background, we aimed to develop a 3D-printed PCL scaffold coated with IGF-1 laden PLGA nanoparticles for cartilage regeneration.

## Methods

### Fabrication of the IGF-1 laden PLGA nanoparticles and composite scaffolds

The double-emulsion (W/O/W) solvent evaporation method was used to produce IGF-1 laden PLGA nanoparticles. Briefly, 20 mg of lyophilized recombinant human insulin-like growth factor-1 (rhIGF-1) were reconstituted in 50 ml of 0.6% (v/v) acetic acid and further diluted to 0.8 ml with 10 m sodium phosphate buffer (pH 6.0) containing 15 mg/ml of Tween 20. This aqueous solution was emulsified with 5 ml of methylene chloride (MC) containing 250 mg of PLGA (75:25) at 13,500 rpm using an ultraturrax for 2 min to form the primary W/O emulsion. This W/O emulsion was then poured into 200 ml of a 2% (w/v) polyvinyl alcohol (PVA)/phosphate-buffered saline (PBS) solution (pH 7.4) and homogenized for 2 min at 13500 rpm. The resulting W/O/W emulsion was stirred at 700 rpm for 4 h at room temperature to allow MC to evaporate. Nanoparticles were recovered after centrifugation at 6000 rpm for 20 min, washed three times with deionized water, freeze-dried, and stored at 4 °C.

Properties of the scaffolds were designed with CAD using Mimics 17.0 software, and the scaffolds were printed by a 3D layer-by-layer fused deposition modeling (FDM) printer (FoChif Tech HTS,China). Briefly, PCL pellets (Mw 80000, Sigma, USA) were melt in a printing chamber at 120 °C and then printed with a lay down pattern of 0°/90°/180° along the z-axis. Thus, a cylinder PCL scaffold model with a diameter of 5 mm and height of 1 mm was produced.

0.3g dopamine (DA, sigma, USA) was dissolved in 150 ml Tris (Sigma, USA) buffer (0.183 g Tris, pH = 8.5) to make a DA solution. PCL scaffolds were immersed in the solution which was stirred (300 rpm) for 24 h at 37 °C protecting from light. Then the scaffolds were washed with deionized water 3 times and dried, and these scaffolds were denoted as PDA-coated PCL scaffolds.

Suspension (0.5%, w/v) of IGF-1 loaded PLGA nanoparticles was dispersed in distilled water, and PDA/PCL scaffolds were immersed in the suspension and stirred (150 rpm) for 2 h. Then the composite scaffolds were dried and the IGF-1 laden PLGA/PDA/PCL scaffolds were obtained.

### Physicochemical characterization

#### Particle size

The nanoparticles were suspended in distilled water, and the nanoparticle diameter was tested using a Malvern particle diameter analyzer (Malvern Instruments, Mastersizer 2000, UK).

#### Loading effciency (LE) and encapsulation effciency (EE)

PLGA nanoparticles (5 mg) were resuspended in NaOH solution (0.04 M, 10 ml) on an orbital shaker at constant 110 rpm gentle shaking at 37 °C for 12 h. A micro bicinchoninic acid (BCA) protein assay kit (Fanbo Biochemicals, Beijing, China) was used for quantitative measurement of IGF-1 loaded in the PLGA nanoparticles. Blank PLGA nanoparticles were used as control. LE and EE of the particles were calculated using the following equations:

LE = mass of IGF-1 contained in PLGA nanoparticles/total mass of PLGA nanoparticles ×100%.

EE = mass of encapsulated IGF-1/total mass of encapsulated and unencapsulated IGF-1 × 100%.

#### Sem

The pure PCL, PDA/PCL, blank PLGA/PDA/PCL and IGF-1 laden PLGA/PDA/PCL scaffolds were sputtered with a thin layer of gold, respectively, and then the surface morphology were tested by scanning electron microscopy (SEM; Hitachi S4800 SEM).

#### Pore size and porosity

The scaffolds were sectioned, embedded in glycol methacrylate (Polysciences, Warrington, PA, USA), and stained with toluidine blue. The stained images were used to calculate pore size and porosity of the scaffolds using pore topology analyzer software (MATLAB, Natick, MA, USA).

#### Compression testing

The compression testing of the scaffold was performed using a mechanical testing machine (INSTRON 5566) with a constant loading rate of 1.0 mm/min, to evaluate the mechanical strength of the prepared scaffolds.

#### Contact angle

The same amount of water was placed on the surface of each scaffold, and then the contact angle was measured using an Automatic Contact Angle Meter instrument (KINO SL200B/K Series, USA).

#### IGF-1 release kinetics of the composite scaffolds

To examine the IGF-1 release profile, IGF-1 loaded composite scaffolds (π × 3^2^ × 1 mm^3^) were placed in a 10000 Da bag filter and incubated in 10 ml of 10 mM phosphate-buffered saline medium (pH 7.4) in a shaking water bath at 37 °C, respectively. Samples were collected every three days for 30 days, and the medium was replaced with an equal volume of fresh buffer. IGF-1 concentrations in the collected samples were measured using an IGF-1 ELISA kit. Measurements were carried out three times.

### Cell culture studies

#### Isolation and culture of chondrocytes and rBMSCs

According to our previous studies (Zhou et al., 2018), rabbit bone marrow-derived mesenchymal stem cells (rBMSC) were obtained the posterior superior iliac spine of the New Zealand white rabbits (4-6 months old) through the bone marrow puncture procedure aseptically, and then were isolated and cultured in a Dulbecco’s modifed Eagle medium (DMEM, Thermo Fisher Scientifc, USA) supplemented with 10% fetal bovine serum (FBS, Thermo Fisher Scientifc, USA) and 1% penicillin–streptomycin (Thermo Fisher Scientifc, USA) at 37 °C under 5% CO_2_. The medium was exchanged every 2–3 days.

Based on our previous study (Li et al., 2019), rabbit chondrocytes were isolated from the cartilage of the proximal side of the joint, washed in PBS, and incubated with collagenase type 1a overnight in DMEM. The cell pellets were centrifuged at 1500 rpm for 5 min and suspended in fresh media. The chondrocytes were then cultivated in DMEM containing 10% (v/v) FBS and 1% (v/v) penicillin–streptomycin at 37 °C under 5% CO_2_.

#### Cells seeding on the scaffolds

The scaffolds were sterilized for 1 h using ultraviolet radiation. At first, fresh media without cells was added to maintain scaffolds in a physiological environment. Samples were then incubated at 37 °C under 5% CO_2_ for 30 min. rBMSCs and chondrocytes were seeded onto the PCL, PDA/PCL, blank PLGA/PDA/PCL and IGF-1-PLGA/PDA/PCL scaffolds, respectively. Media was subsequently changed daily, and samples were cultured under standard conditions.

#### CCK-8

For cell proliferation assays, chondrocytes and rBMSCs were seeded at a density of 5 × 10^4^ cells/ml in wells of a 96-well plate containing scaffold samples and cultured for 1, 3, 5, 7 d, respectively. Subsequently, the scaffolds with cells were transferred to a new culture plate and were incubated in 10% CCK-8 solution in a 37 °C and 5% CO_2_ incubator for 1.5 h, Cell activity was evaluated using a cell counting kit (CCK-8, Sigma-Aldrich) according to the manufacturer’s instructions. The absorbance at 450 nm of the sample solutions was measured by a microplate reader. (Synergy 2; BioTek, USA).

#### Live/dead

The viability of rBMSCs and chondrocytes seeded onto the different scaffolds was assessed using a Live/Dead kit (Life Technologies, USA). Cells at a density of 5 × 10^4^ cells/ml were added to wells of a 96-well plate containing the scaffolds and cultured for 1 day. Then, the scaffolds were washed with PBS and the staining solution was prepared according to the manufacturer’s instructions of the Live/Dead kit. Then, 400 µl staining solution was added to the scaffolds and incubated for 30 min at 37 °C in the dark. The samples were then washed in PBS buffer solution twice. Images were captured using a confocal microscope (Leica TCS SP2 confocal microscope; Leica, Mannheim, Germany). Live cells were stained green and dead cells were stained red.

#### MTT

The Vybrant^®^ MTT Cell Proliferation Assay Kit (Thermo Fisher Scientifc, USA) was used to assess biocompatibility of the scaffolds and cell proliferation 1, 3, 5, and 7 days after seeding. We added 0.25% trypsin to BMSC/scaffolds and chondrocytes/scaffolds composites to get the cells from scaffolds. Then, the solutions were centrifuged at 1200 rpm for 5 min. The supernatant was then removed and 100 μl phenol-free DMEM and 10 μl of MTT reagent were added to the cell pellets. Cell pellets were incubated at 37 °C and 5% CO_2_ for 4 h. Then, 25 μl of dimethyl sulfoxide (DMSO) was added, and cell pellets were incubated for another 20 min. The solution was transferred to 96-well disks. Absorbance readings at 570 nm were recorded using a Benchmark Plus microplate spectrophotometer (Bio-Rad, Tokyo, Japan).

#### Glycosaminoglycan (GAG)

Based on our previous studies (Li et al., 2019), GAG content of each chondrocytes/scaffolds and rBMSCs/scaffolds composites at 7 and 14 days after seeding were analyzed by sulfated glycosaminoglycan assay (Biocolor, UK). An ultraviolet spectrophotometer was used to quantitatively determine GAG content by measuring the absorption at 656 nm, and the values of GAG content were calculated based on a standard curve. All experiments were normalized to the total protein content determined from the BCA Protein Assay Kit (Beyotime, China), which were independently performed in triplicate.

#### Protein expression evaluation

Western blot (WB) was performed after seven days of in vitro culture, and the protein samples were extracted from the cell/scaffold composites and homogenized in lysis buffer containing 50 mM Tris-HCl (pH 7.5), 200 mM NaCl, 10 mM CaCl_2_, 0.02% NaN_3_, and 0.05% Triton X-100, according to our previously published method (Xu et al., 2015). The total protein level in the supernatants was measured using the BCA Protein Assay Kit (Thermo Scientific, Rockford, IL, USA). 10% Tris–HCl gel was applied to separate the proteins by electrophoresis. Next, the proteins were transferred to PVDF membranes for 45 min at 300 mA. The PVDF membranes were blocked with 5% BSA at room temperature for 1 h and the membranes were incubated with the primary antibodies at 4 °C overnight. Then, the blots were washed with Tris-buffered saline/Tween-20 (TBST) and incubated in the secondary antibodies for two hours. The antibodies applied were as follows: primary antibodies (SOX-9, type II collagen (COL-II), aggrecan (ACAN), glyceraldehyde-3-phosphate dehydrogenase (GAPDH) (Abcam, UK)) and corresponding HRP-conjugated secondary antibodies. Enhanced chemiluminescence was used to detect antibodies. Protein expression normalized to GAPDH was analyzed using Image J software (National Institutes of Health, USA).

#### Gene expression evaluation

To evaluate the mRNA transcript levels of chondrocytes specific genes (SOX-9, COL-II and ACAN), chondrocytes and rBMSCs were cultured on the scaffolds for 7 and 14 days and processed for total RNA extraction by using a trizol kit (Ambion, CA) according to the manufacturer’s instructions, and the concentration of RNA was determined at 260 nm with a multifunction microplate reader (Synergy 2; BioTek, USA). The cDNA was reverse-transcribed using All-In-One RT MasterMix (ABM, CA), and quantitative real-time polymerase chain reaction (qRT-PCR) was performed using EvaGreen 2 × qPCR MasterMix (ABM, CA) with a Light Cycler 480 II (Roche, CHE). To study the underlying mechanism of IGF-1 promoting chondrogenesis, the expression level of HIF-1α was examined. Chondrocytes and rBMSCs were cultured on the scaffolds for 24 h, and then qRT-PCR analysis was performed as described above. GAPDH was used as the housekeeping gene for normalization. Primer sequences are listed in Table 1.

**Table 1.** Forward and reverse primer sequences used for reverse transcriptase PCR.

Gene	Forward primer	Reverse primer
GAPDH	5′-TCACCATCTTCCAGGAGCGA-3′	5′-CACAATGCCGAAGTGGTCGT-3′
SOX-9	5′-GGTGCTCAAGGGCTACGACT-3′	5′-GGGTGGTCTTTCTTGTGCTG-3′
COL-II	5′-AACACTGCCAACGTCCGAT-3′	5′-CTGCAGCACGGTATAGGGA-3′
ACAN	5′-AGGTCGTGGTGAAAGGTGTTG-3′	5′-GTAGGTTCTCACGCCAGGGA-3′
HIF-1α	5′-CCATGTGACCATGAGGAAAT-3′	5′-CGGCTAGTTAGGGTACACTT-3′

### Statistical analysis

All statistical analyses were performed using SPSS 18.0 statistical software and all analysis was expressed as means ± standard deviation (SD). Multiple sets of data were analyzed using one-way ANOVA with a post hoc test, and differences between 2 groups were analyzed by t test. *p* < .05 was considered statistically significant.

## Results

### Characteristics of the IGF-1 laden nanoparticles and composite scaffolds

**Figure 1.**
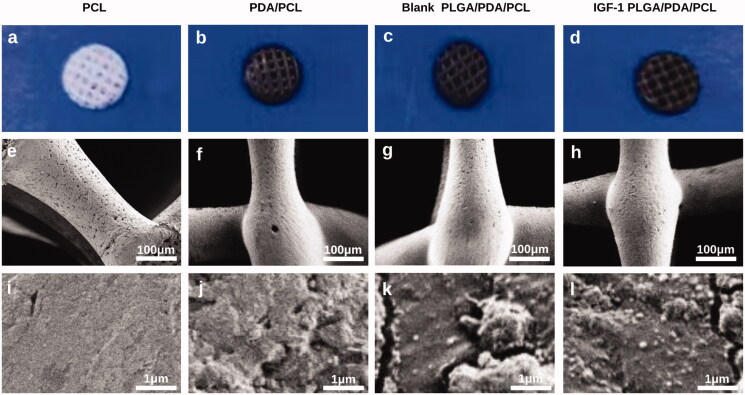
**(**a–d) General appearance of the PCL, PDA/PCL, blank PLGA/PDA/PCL, IGF-1 PLGA/PDA/PCL scaffolds. (e–l) SEM images of the PCL, PDA/PCL, blank PLGA/PDA/PCL, IGF-1 PLGA/PDA/PCL scaffolds.

After coating of the PCL scaffolds with PDA, the PCL scaffold color changed from white to black (Figure 1(a,b)). With the adherence of the PLGA nanoparticles, no further change in the general color occurred (Figure 1(c,d)). Under SEM at low magnification, the prepared scaffolds with pores arranged in the 0°–90°–180° quadrate pattern showed no obvious differences in morphology or structure (Figure 1(e–h)). At high magnification, PCL scaffolds showed a smooth surface (Figure 1(i)), while PDA/PCL scaffolds showed small, evenly distributed granules (Figure 1(j)), and PLGA/PDA/PCL scaffolds had PLGA nanoparticles attached to the small granules (Figure 1(k,l)). There showed no significant difference in morphology between blank PLGA/PDA/PCL scaffold and IGF-1 laden PLGA/PDA/PCL scaffold at high magnification.

**Figure 2.**
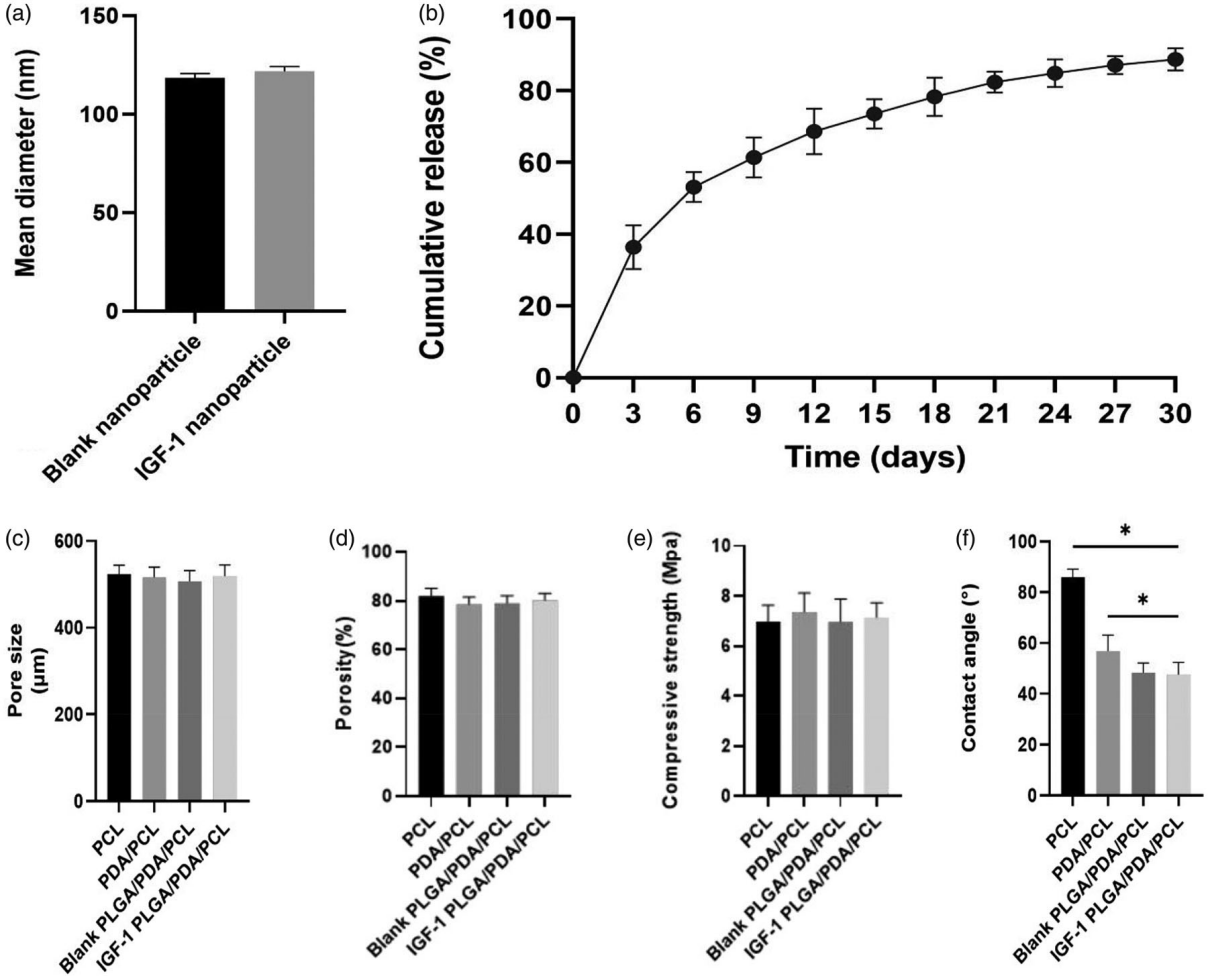
(a) Mean diameter of the prepared nanoparticles. (b) IGF-1 release kinetics of the fabricated IGF-1 laden PLGA/PDA/PCL composite scaffold. (c) Pore size of each scaffolds. (d) Porosity of each scaffold. (e) Compressive strength of each scaffolds. (f) Contact angle of each scaffold. *p < .05.

The mean diameter of the nanopartsicle was 121.77 ± 2.39 nm, which showed no difference between the blank PLGA nanoparticles and IGF-1 laden PLGA nanoparticles (Figure 2(a)). Moreover, the LE of IGF-1 in PLGA nanoparticles achieved was about 5.53 ± 0.12%, and the EE was about 61.26 ± 2.71%.

The IGF-1 loading amount of the IGF-1 laden PLGA/PDA/PCL scaffolds was 141.17 ± 11.66 μg. From the release curves of the scaffolds shown in Figure 2(b), an initial burst release of IGF-1 was obvious, and the majority of drug (36.37%) was released within 3 days, followed by a slower, more sustained release for all drug-loaded nanoparticles PLGA/PDA/PCL scaffolds, with a cumulative release rate of 88.71% at 30 days.

Figure 2(c) showed the regular porous micro-structure of the scaffold, and the pore size of the PCL,PDA/PCL,blank PLGA/PDA/PCL and IGF-1 PLGA/PDA/PCL composite scaffolds were 523.81 ± 20.66 μm, 516.45 ± 23.21 μm, 507.25 ± 24.39 μm, and 518.57 ± 26.17 μm, respectively, and the porosity of the PCL, PDA/PCL, blank PLGA/PDA/PCL and IGF-1 PLGA/PDA/PCL composite scaffolds were 81.87 ± 3.17%, 78.58 ± 2.86%, 78.98 ± 3.13%, and 80.12 ± 2.90%, respectively (Figure 2(d)), which showed no significant difference.

As shown in Figure 2(e), The compressive strength of the scaffolds were 6.98 ± 0.65 MPa, 7.35 ± 0.77 MPa, 6.96 ± 0.92 MPa and 7.14 ± 0.59 MPa, respectively, which showed no significant difference.

The contact angles of PCL, PDA/PCL, blank PLGA/PDA/PLGA and IGF-1 laden PLGA/PDA/PLGA scaffolds were 85.92 ± 3.17°, 56.77 ± 6.32°, 48.29 ± 3.86° and 47.53 ± 4.81°, which showed significant difference (Figure 2(f)).

### Cell survival, viability and proliferation on all prepared scaffolds

**Figure 3.**
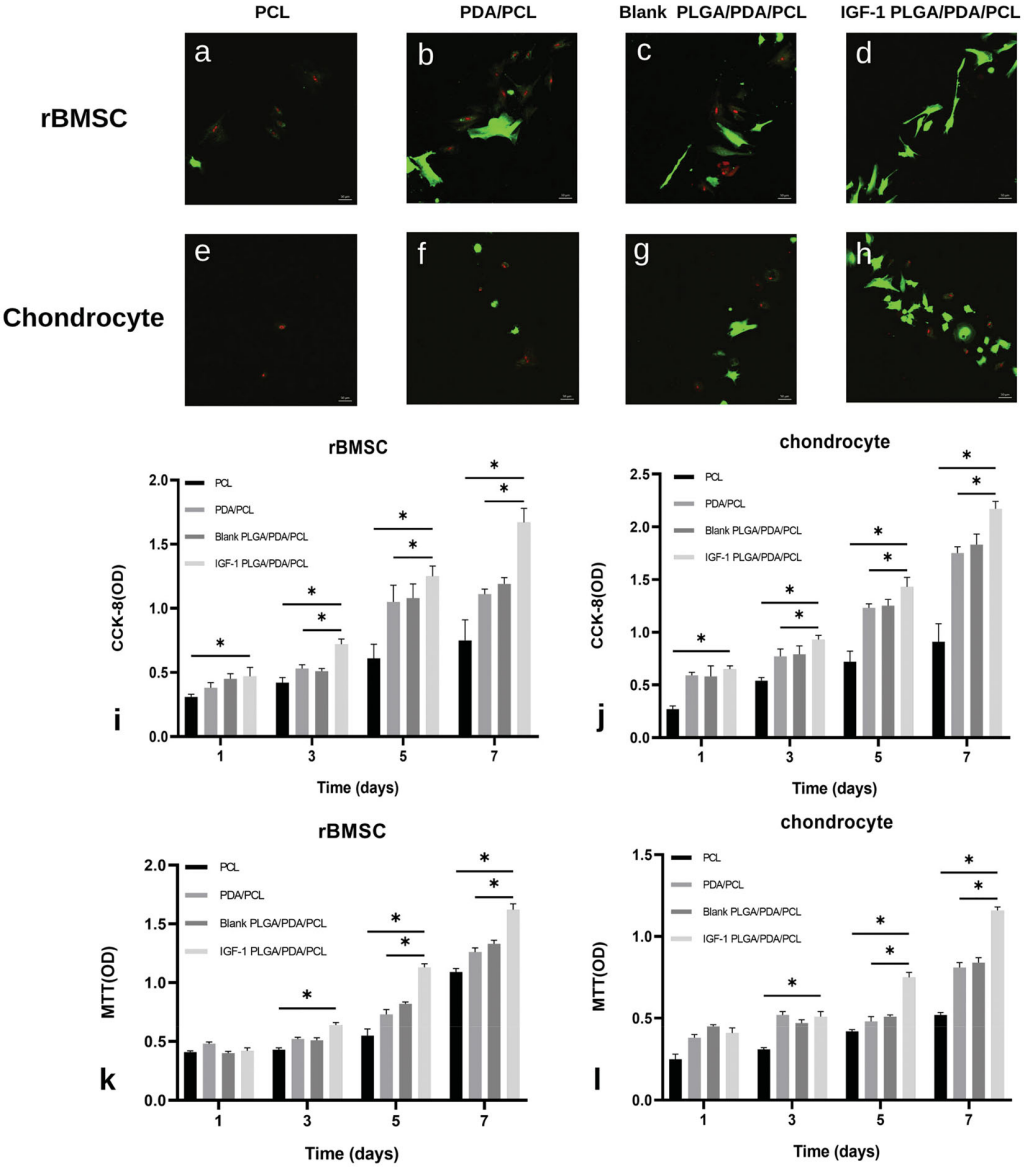
**(**a–h) Confocal images of live/dead staining at day 1 of culture on the PCL, PDA/PCL, blank PLGA/PDA/PCL, IGF-1 PLGA/PDA/PCL scaffolds, showing live cells (green) and dead cells (red). (i,j) Proliferation of rBMSCs and chondrocytes on each scaffold type as measured by CCK-8 assay. (k,l) Biocompatibility analysis of the scaffolds by MTT. *p < .05.

Live/dead staining of rBMSCs seeded on the different scaffolds after 1 day in culture revealed that almost no dead rBMSCs existed on IGF-1 laden PLGA/PDA/PLGA scaffolds, and more cells were present on the PDA/PCL and PLGA/PDA/PCL scaffolds than on the pure PCL scaffold (Figure 3(a–d)). Similarly, IGF-1 laden PLGA/PDA/PCL scaffolds had obviously more chondrocytes attached than the other three scaffolds (Figure 3(e–f)).

The CCK-8 assay showed that the increase in the number of cells over time with all scaffold samples indicated that all of the prepared scaffolds were nontoxic to the cells (Figure 3(i,j)). On day 1,3,5,7, the OD values, representing cell number, increased with increasing culture time in all groups, and for the IGF-1 laden PLGA/PDA/PCL scaffold group displayed a significantly higher OD value than that for the other three groups (Figure 3(i,j)).

The MTT assay results showed higher cell proliferation in IGF-1 laden PLGA/PDA/PCL scaffolds at 5 and 7 days compared to other three scaffolds (Figure 3(k,l)).

### GAG assay

**Figure 4.**
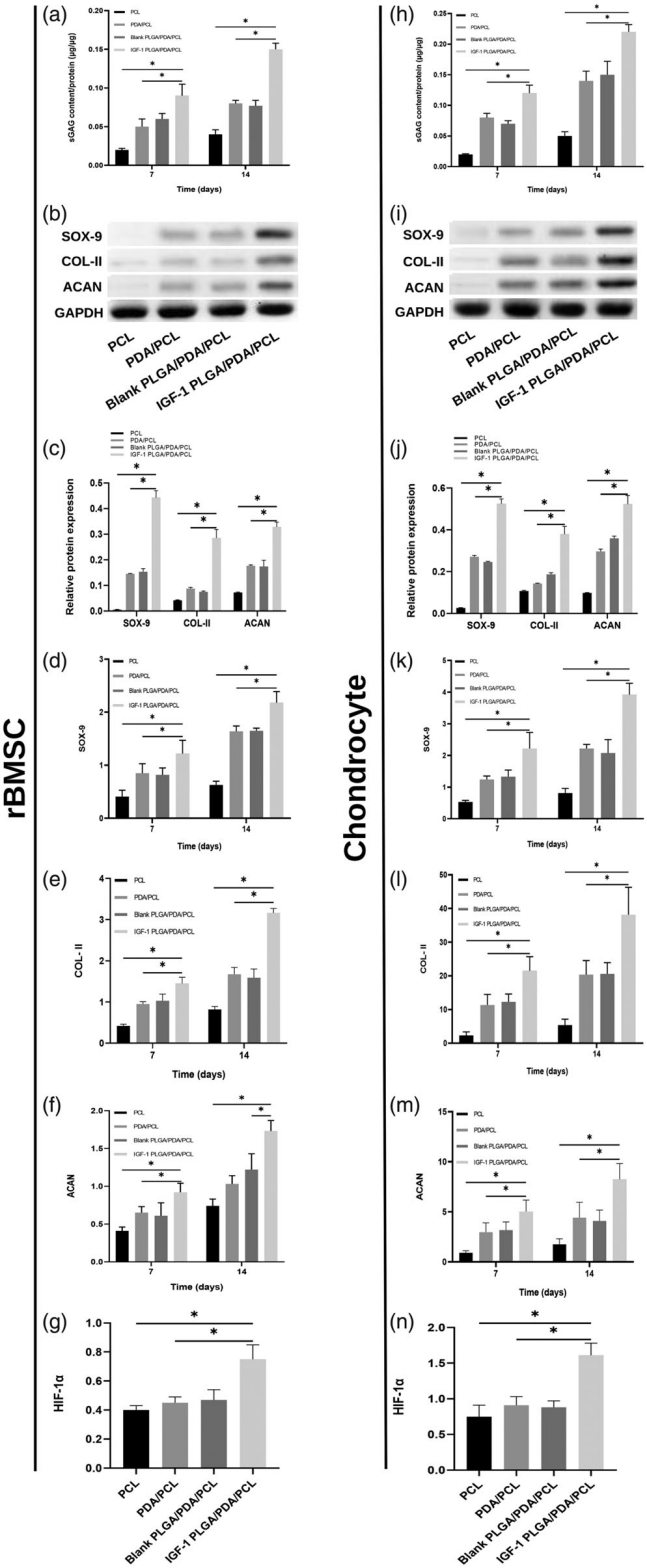
**(**a,h) GAG accumulation of rBMSC and chondrocytes on day 7 and 14 normalized to total protein concentration. (b,c,i,j) Grayscale maps and relative chondrogenic protein expression of SOX-9, COL-II, and ACAN after 7 days normalized to GAPDH expression by WB analysis. (d–f,k–m) Relative chondrogenic gene expression of SOX-9, COL-II, and ACAN on day 7 and 14 normalized to GAPDH expression by qRT-PCR. (g,n) The expression of HIF-1α gene in each scaffolds cultured rBMSC and chondrocytes by qRT-PCR. *p < .05.

The results of GAG content were shown in Figure 4, normalized to total protein content of rBMSCs and chondrocytes cultured on the 4 kinds of scaffolds. Both in rBMSCs and chondrocytes, the GAG contents/protein ratios increased with time in all scaffolds and was higher in IGF-1 PLGA/PDA/PCL scaffolds than in other three scaffolds (Figure 4(a,h)).

### Protein and gene expression evaluation

As shown in Figure 4, WB results showed significantly increased expression of SOX-9, COL-II and ACAN in BMSCs and chondrocytes cultured in IGF-1 PLGA/PDA/PCL scaffold group as compared to other three groups (Figure 4(b,c,i,j)). And the results were in good agreement with those of the qRT-PCR that all chondrogenic gene expression markers (SOX-9, COL-II and ACAN) showed increasing trends in all scaffolds, while the expression level was significantly higher in IGF-1 laden PLGA/PDA/PCL scaffolds on days 7 and 14 compared to other groups (Figure 4(d–f,k–m)). The highest protein and gene expression in IGF-1 laden PLGA/PDA/PCL scaffolds correspond to the most significant GAG content result in this group.

To examine the underlying mechanism of IGF-1 promoting chondrogenesis, the HIF pathway related gene was analyzed. qRT-PCR results showed that the expression of HIF-1α was significantly elevated in IGF-1 laden PLGA/PDA/PCL cultured group as compared to pure PCL, PDA/PCL and blank PLGA/PDA/PCL groups (Figure 4(g,n)).

## Discussion

Various in vitro and in vivo studies have focused on scaffolds for cartilage regeneration and have achieved some success (Fisher et al., 2014), but long-term results are still controversial, as chondrocyte phenotype alteration and fibrocartilage formation occur (Ng et al., 2017). Improvement of scaffolds is needed to provide a better environment for chondrogenesis.

3D-printed PCL scaffolds with good bio-function and cells-scaffold interaction have been widely used in cartilage engineering (Zhang et al., 2017; Dao et al., 2019). However, PCL has poor cell affinity due to its hydrophobic surface and lack of cell biological recognition (Tallawi et al., 2015). It has been suggested that the PDA coating not only changes the hydrophobicity of PCL, but also significantly increases cell adhesion to the scaffold (Jo et al., 2013). Lower water contact angle showed better hydrophilicity, as rBMSCs and chondrocytes were cultured in an aqueous environment (Li et al., 2019). Our results showed that the incorporation of PDA and PLGA nanoparticles into the PCL scaffold significantly decreased the water contact angle of the pure PCL which can provide a much better biomimetic extracellular matrix (ECM) microenvironment. While the pore size, porosity and compressive strength were retained, which provided an appropriate pore space to facilitate cell infiltration and nutrition supply, and was suitable to withstand the forces on the joint (Murphy et al., 2016; Li & Zhang, 2018; Li et al., 2019). The CCK-8 assay and live/dead test showed that more cells were adhered to the PLGA/PDA/PCL and PDA/PCL scaffolds than to the pure PCL scaffolds, also indicated by higher expression of cell adhesion molecules, which was consistent with the bioadhesive capacity of PDA (Zhou et al., 2018; Li et al., 2019).

Despite a growing interest in the development of composite scaffolds for drug delivery, there is a need for a carrier to provide a controlled release of bioactive factors (Wang et al., 2018). The nanocarrier material PLGA which has a good bio-security has gained much attention (Wang et al., 2013). In the present study, the release profile of IGF-1 laden PLGA/PDA/PCL composite scaffold showed a sharp initial burst release for 3 days, followed by a slowly sustained release for over 30 days, which indicated that it can provide a sustained chondrogenic induction effect during the process of cartilage healing. The burst phase may be caused by the rapid diffusion of surface-bound drug, whereas the lag phase may be determined by the dissolution of PLGA materials and release of the drug from the inner part of the nanoparticles (Hickey et al., 2002; Dawes et al., 2012).

In addition, The CCK-8, MTT and Live/Dead analysis revealed that the released IGF-1 had no cytotoxicity, and showed that more cells were adhered to the IGF-1 laden PLGA/PDA/PCL scaffolds than the other scaffolds. It has been demonstrated that IGF-1 is an important factor in preservation of articular cartilage and can stabilize the chondrogenic potential of chondrocytes (Claassen et al., 2011). An IGF-1 deficiency is responsible for the degree of severity of osteoarthritic lesions (Ekenstedt et al., 2006), and IGF-1 is the cartilage matrix degrading enzyme that down-regulates the IL-1-stimulated mRNA expression of MMP1, −3, −8 and −13 in human chondrocytes (Hui et al., 2001; Claassen et al., 2011).

Our study showed that the GAG content in the IGF-1 PLGA/PDA/PCL group was significantly higher than in the other groups, and WB and qRT-PCR results showed that the IGF-1 laden PLGA/PDA/PCL scaffold group had the highest chondrogenic specified proteins and genes expression of SOX-9, COL-II and ACAN, which indicated its higher chondrogenic capacity than the other scaffolds and the enhancing effect on chonldrogenesis of IGF-1. It has been demonstrated (Li et al., 2019) that SOX-9 can upregulate the expression of COL-II and ACAN, and the enhanced expression of COL-II and ACAN provides better new matrix synthesis and deposition for cartilage repair, of which the main part is GAG. In our study, the highest GAG content and significantly increased COL-II expression in IGF-1 laden PLGA/PDA/PCL scaffold group indicated a massive accumulation of GAG and possibly enhanced synthesis of collagen II, and also indicated that the microenviroment around the composite scaffold was remodeled with these newly formed chondrocyte ECM proteins, subsequently providing a suitable environment for proliferation and differentiation of chondrocytes and rBMSC, ultimately preventing osteoarthritis deterioration, and assisting cartilage regeneration as reported by previous studies (Morgan et al., 2006; Li et al., 2012; Eslaminejad et al., 2013; Deng et al., 2017; Tanthaisong et al., 2017; Li et al., 2019).

It has been reported that the significantly increased chondrogenic expressions (SOX-9, COL-II and ACAN) might be regulated by the activated hypoxia-inducible factor pathway and could affect the cell behaviors and support energy regeneration of chondrocyte and rBMSC (Eslaminejad et al., 2013; Li et al., 2019). However, HIF pathway is closely related to the chondrocytes maturation and cartilage regeneration (Gelse et al., 2008; Bouaziz et al., 2016; Deng et al., 2017). To verify this hypothesis, the expression of HIF-1α in chondrocytes and rBMSCs cultured in these scaffolds were carefully analyzed. It was found that the expression of HIF-1α in IGF-1 laden PLGA/PDA/PCL scaffold group was markedly enhanced as compared to other groups. As revealed in previous reports, the activated HIF pathway was able to stimulate the expression of ACAN, COL-II and SOX-9 production to support energy regeneration and matrix synthesis for cartilage regeneration (Pfander et al., 2003; Komatsu & Hadjiargyrou, 2004; Bouaziz et al., 2016), which was consistent with our results. Consequently, it is probable that the potential mechanism for IGF-1 promoting chondrogenesis may be closely related to the activated hypoxia-inducible HIF pathway.

In conclusion, the IGF-1 laden PLGA/PDA/PCL scaffold was successfully fabricated in our study, and our results showed its better capacity of chondrogenesis, which may be a better method for sustained IGF-1 administration and a promising scaffold for cartilage tissue engineering.
